# Fractal aggregation kinetics contributions to thermal conductivity of nano-suspensions in unsteady thermal convection

**DOI:** 10.1038/srep39446

**Published:** 2016-12-20

**Authors:** Jize Sui, Peng Zhao, Bandar Bin-Mohsin, Liancun Zheng, Xinxin Zhang, Zhengdong Cheng, Ying Chen, Goong Chen

**Affiliations:** 1 School of Energy and Environmental Engineering, University of Science and Technology Beijing, Beijing 100083, China; 2 School of Mathematics and Physics, University of Science and Technology Beijing, Beijing 100083, China; 3 School of Chemistry, Beijing Normal University, Beijing 100875, China; 4 Department of Mathematics, King Saud University, Riyadh, Saudi Arabia; 5 Artie McFerrin Department of Chemical Engineering, Texas A&M University, College Station, Texas 77843, USA; 6 Guangdong Provincial Key Laboratory of Functional Soft Condensed Matter, Materials and Energy School at Guangdong University of Technology, Guangzhou 510006, China; 7 Department of Mathematics and Institute for Quantum Science and Engineering, Texas A&M University, College Station, TX 77843, USA; 8 Science Program, Texas A & M University at Qatar, Education City, Doha, Qatar

## Abstract

Nano-suspensions (NS) exhibit unusual thermophysical behaviors once interparticle aggregations and the shear flows are imposed, which occur ubiquitously in applications but remain poorly understood, because existing theories have not paid these attentions but focused mainly on stationary NS. Here we report the critical role of time-dependent fractal aggregation in the unsteady thermal convection of NS systematically. Interestingly, a time ratio *λ* = *t*_*p*_/*t*_*m*_ (*t*_*p*_ is the aggregate characteristic time, *t*_*m*_ the mean convection time) is introduced to characterize the slow and fast aggregations, which affect distinctly the thermal convection process over time. The increase of fractal dimension reduces both momentum and thermal boundary layers, meanwhile extends the time duration for the full development of thermal convection. We find a nonlinear growth relation of the momentum layer, but a linear one of the thermal layer, with the increase of primary volume fraction of nanoparticles for different fractal dimensions. We present two global fractal scaling formulas to describe these two distinct relations properly, respectively. Our theories and methods in this study provide new evidence for understanding shear-flow and anomalous heat transfer of NS associated non-equilibrium aggregation processes by fractal laws, moreover, applications in modern micro-flow technology in nanodevices.

Colloidal suspensions with dispersed nanosized materials (nanoparticles, nanotubes, nanowires) named “nano-suspensions” (NS) are new thermal transport medium with great potential applications in many fields, such as micro heat exchangers, micro-electronics, chemical engineering, and aerospace^1^,^2^. A number of experimental measurements and numerical simulations have been reported on the heat conduction enhancements of these nano-suspensions containing small volume fraction of nanoparticles (NP) (Particle loading <0.1) relative to the base fluid^3^,^4^.

The NS exhibit promising thermophysical properties, nevertheless, the study of certain anomalous mechanisms in NS has not reached concrete conclusions. NS, also named “nanofluid”^5^, are not a simple mixture of liquid and nanoparticles owing to their complex solid/liquid surface interactions (Interfacial phenomenon) and interparticle attraction. Therefore, some anomalous heat transfer behavior can be affected profoundly by particle-liquid interfacial ordered layer (IOL)^6^,^7^,^8^ and interparticle aggregations^9^,^10^,^11^.

Various thermal conductivity models, as key factors for the theoretical characterization of the real heat transfer performance of NS, were proposed to predict the real thermal behavior of NS. But many such models are not in agreement with experimental data. In general, almost all models are based on the classical mean-field theory of Maxwell hard-sphere particles^12^


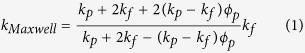


where *ϕ*_*p*_ is volume fraction of primary NP, and *k*_*Maxwell*_, *k*_*p*_ and *k*_*f*_ are the thermal conductivity of NS, NP and base fluid, respectively. The transmission electron microscopy (TEM) in Fig. 1 illustrates the typical aggregation in NS of Al_2_O_3_/water (left) and CuO/water (right), respectively^13^. In a recent paper^14^, we presented a multilevel equivalent agglomeration (MEA) thermal conductivity model for stationary NS, which have achieved successful theoretical predictions. The highly consistent predictions with classical data (by Lee *et al*.^13^) are presented in that paper.

It is common in NS that nanoparticles tend to aggregate with the time due to its high surface potential energy (surface-activity), in consequence, there is more difficulty in probing the thermal performance of NS with dispersion of fully irregular clusters. Weitz, *et al*.^15^ found that the scaling behavior in diffusion-limited aggregation in colloidal solution depends on both the average cluster size and initial concentration with the evolution of time^15^, and the fractal dimension 1.75 ≤ *d*_*f*_ ≤ 2.05 for gold aggregates was displayed^16^. Subsequently, scaling law in terms of volume fraction of aggregates, the contained number of nanoparticles per cluster and fractal dimensions of clusters size were reported in refs 17, 18, 19. The scaling law relationships between the characteristic geometric size of aggregates and time was established by Hanus *et al*.^20^ in which the aggregate characteristic time *t*_*p*_ was proposed explicitly. Prasher *et al*.^21^ investigated experimentally the distribution of *t*_*p*_ for nano-alumina suspensions and a large time-domain independence of nanoparticles radius, temperature and pH was presented.

Unfortunately, up to now, most researchers have focused on the investigation of stationary NS. It is unclear how the non-equilibrium aggregation alters the heat transfer properties of kinetic NS induced via shear flow or thermal convection often encountered in modern nanotechnology applications^22^. In modern technology, nano-suspensions used in most engineering applications should meet the various thermal convection requirements, for instance, the shear flow and heat transfer of nano-suspensions in biological tissue, DNA replication and amplification via natural convection processes etc.^23^,^24^. Furthermore, enhanced heat transfer efficiency in other heat exchange devices using diluted NS, such as solar collectors, cooling systems etc., is often accompanied by the generation of thermal convections^25^,^26^. The aggregate morphology described by quasi-fractal has been modeled in altering the convection heat transfer performance in nanofluids, and some specialized models for convective heat transfer coefficient, dynamic viscosity etc. were proposed^27^, but there is no more exploration for unsteady convection heat transfer in nano-suspensions governed by N-S equations with adopting the perception on fractal aggregation kinetics.

As a result, the aggregate formation in unsteady flowing NS should change drastically relative to stationary ones. With such an understanding, nevertheless, existing investigations have not yet demonstrated how important a role non-equilibrium aggregates play on the thermal transport of NS. In this study, we report a novel physical process that time-dependent fractal aggregation kinetics affects the unsteady thermal convection boundary layer of NS. The analysis both of convection flow and temperature distributions of NS in the boundary layer are considered to obtain what we regard as interesting results by incorporating non-equilibrium fractal aggregation mechanisms.

## Theoretical description and formula

**Figure 2:**
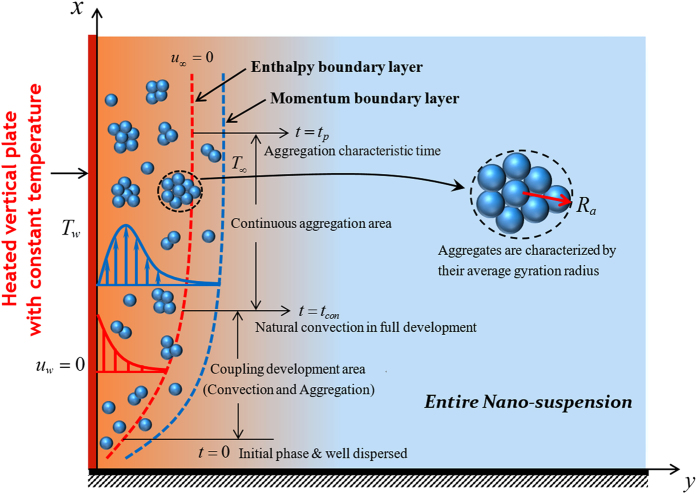
Cartoon of dynamic NS with aggregation. Schematic cross section of unsteday thermal convection boundary layer system of nano-suspensions (NS) incorporating nonequilibrium aggregation processes and Cartesian coordinates system.

According to natural convection boundary layer (NCBL) theories^28^ (Zheng and coauthors have carried out some studies^29^,^30^,^31^), there are large velocity gradient (shear flow) and temperature gradient (heat transfer) in NCBL and they are both coupled. Consider a two-dimension unsteady laminar natural convection flow and heat transfer of the nano-suspensions on a heated vertical plate as shown in Fig. 2. The velocity and temperature boundary layers both develop over time near the plate along the x-axis. Herein, *T*_*w*_ denotes the constant temperature of the plate surface and *T*_∞_ the ambient temperature of NS. It is assumed that the homogeneous NS is at rest at the initial time *t* = 0 and no mass transfer occurs over all the time. More importantly, the base fluid has large magnitude in altering the rheology properties of NS, and the consensus is that the rheology properties have little changes from Newtonian characteristics with particles loading *ϕ*_*p*_ ≤ 0.1 for water base fluid with a certain range of shear rate, in spite of the dynamic viscosity and effective thermal conductivity of NS are dependent on the particles volume fraction apparently. The NS will exhibit very complex performances, perhaps shear-thinning or shear-thicken, once the particles loading is higher (>0.1).

It is applicable that the rheology and heat conducting constitutive models of NS with maximum particle loading 0.1 are addressed as Newtonian models within the circumstance of natural convection boundary layer^29^,^30^,^31^. Then, the governing equations of this system can be written as


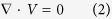



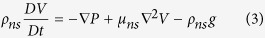



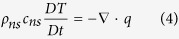


where *V*(*u, v*) is the two-dimension velocity vector with *u* along the x-axis and *v* along the y-axis, respectively, *g* the gravitational acceleration, *T* the temperature and *t* the time. *ρ*_*ns*_ and *c*_*ns*_ are density and specific heat of NS, respectively. 
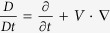
 is the material derivative. 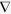 and 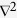 are the gradient and the Laplace operator, respectively. The pressure gradient is 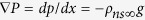 with 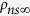 the density of the ambient NS. By invoking the Boussinesq approximation, we have 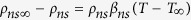, with *β*_*ns*_ the thermal expansion coefficient of NS. The modified Fourier heat conduction law for NS is employed as 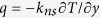 with *k*_*ns*_ the thermal conductivity of NS, subject to the boundary conditions


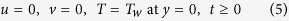



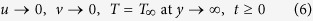


We now investigate the time-dependent fractal aggregation kinetic model by introducing two important scaling laws as^20^,^21^


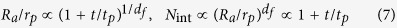


where *r*_*p*_ is the radius of primary nanoparticle, *R*_*a*_ an average gyration radius of clusters (shown in Fig. 2), *t*_*p*_ the aggregate characteristic time (aggregation time constant), *N*_int_ the average number of primary nanoparticles in a single aggregate and *d*_*f*_ the fractal dimension of the aggregates. Often chosen are *d*_*f*_ ≈ 2.5 for reaction-limited aggregation and *d*_*f*_ = 1.8 for diffusion-limited aggregation according to DLCA^21^ and Eq. 7 is reported to be valid for DLCA clusters rather than for RLCA clusters. Moreover, 1.8 ≤ *d*_*f*_ ≤ 2.3 was manifested to be suitable for aggregated nano-alumina suspensions^32^. One can conclude the clusters size distribution from Eq. 7 with depending on the hold time and fractal dimensions.

Furthermore, if *ϕ*_*p*_ is the primary volume fraction of NP, *ϕ*_int_ denotes the primary NP volume fraction inside aggregates and *ϕ*_*a*_ the volume fraction of aggregates in the entire NS, we have the relation *ϕ*_*p*_ = *ϕ*_int_*ϕ*_*a*_^20^,^21^. With the hard-sphere and homogenization assumptions that the aggregates formed by primary nanoparticles are in uniform size (a sphere with radius *R*_*a*_), one can derive another important scaling law according to Eq. (7) as


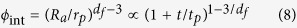


Note that *ϕ*_*a*_ = *ϕ*_*p*_/*ϕ*_int_ signifies the formation process of time-dependent fractal aggregation with time evolution. Subsequently, we obtain the density, effective heat capacity and effective thermal conductivity of fractal aggregates by considering the percolation effects in clusters, respectively:


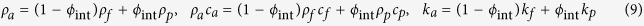


where, subscript “*f*” is for the based fluid, “*a*” for aggregates and “*p*” for nanoparticles. Non-equilibrium aggregation occurs in time 0 ≤ *t* ≤ *t*_*p*_, otherwise an equilibrium state of aggregates form if *t* > *t*_*p*_. Consequently, we obtain the renovated Maxwell model by considering fractal aggregation effects, once Eq. (8) is introduced, as


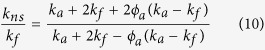


Here the enhanced thermal conductivity of dynamic NS is described by means of this modified model with the focus on time-dependent fractal laws prominently, which is regarded as underlying mechanism in thermal convection flow rather than other factors commonly are involved for stationary NS^14^. Based on Eq. (9), we derive the effective physical parameters of flowing nano-suspensions as 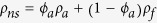, 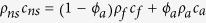 and dynamic viscosity is suggested by the Brinkman model^33^ 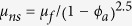.

Furthermore, we seek similarity solutions of Eqs (2,3,4) to simplify the process of solving partial differential equations systems^29^,^30^,^31^. The elaborated similarity transformation for unsteady boundary layer thermal convection including the stream function *ψ*, similarity variables and dimensionless temperature function are shown as


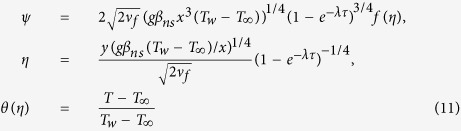


where *ν*_*f*_ = *μ*_*f*_/*ρ*_*f*_ is kinematic viscosity of base fluid, *f*(*η*) the dimensionless stream function and *τ* = *t*/*t*_*p*_ the dimensionless time, respectively. Substituting Eq. (11) into Eqs (2,3,4,5,6), in view of (*u, v*) = (*ψ*_*y*_, −*ψ*_*x*_), we derive the following coupled nonlinear equations as


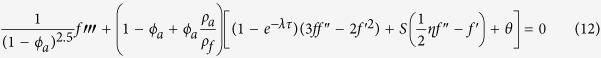



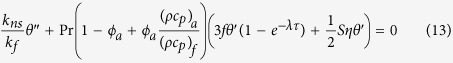


with boundary conditions


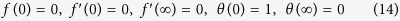


where Pr is the Prandtl number of the base fluid (water), and 
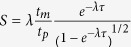
 is the dimensionless unsteady parameter. One then obtains 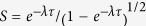 by adopting *λ* = *t*_*p*_/*t*_*m*_ (*t*_*m*_ = *L*/*u*_*m*_ is mean time). Ensuing, we obtain dimensionless velocity 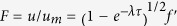, where 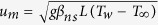 is average velocity at a reference length *L* in NCBL^29^,^30^. Obviously, *S* → 0 as the dimensionless time *τ* → ∞, i.e., the unsteady model will be reduced to the steady model.

The major engineering parameters for this problem are the skin friction coefficient 
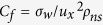
 and the Nusselt number 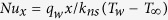, where 
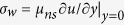
and 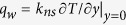 are the surface shear stress and heat flux along the vertical plate. In view of the similarity variables above, we define the local skin friction and local Nusselt number, respectively as


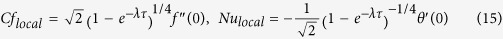


## Discussion

### Slow and Fast aggregation

The characteristic time ratio parameter *λ* = *t*_*p*_/*t*_*m*_ is closely related to the aggregates formation processes and thermal convection flow, which can be used to characterize the slow aggregation (SA) and fast aggregation (FA) processes relative to thermal convection processes.

As reported by Prasher *et al*.^21^, the aggregate characteristic time in a broad range 10 < *t*_*p*_ < 10^5^ seconds is corresponding to the primary nanoparticles size around 5~10 nm at 55 °C, or larger for aqueous nano-alumina suspension with hard sphere shape nanoparticles, which is also chosen as the working NS in this study. Prandtl number Pr ≈ 3.2 (water at 55 °C), initial volume fraction *ϕ*_*p*_ = 0.05 and *d*_*f*_ = 1.8 and other important thermophysical properties presented in Table 1 are suitable for calculations. Eqs (12,13,14) are solved numerically by employing a shooting technique with a high-efficiency fourth order Runge-Kutta algorithm.

Here, we may set an empirical average convection time *t*_*m*_ = 66.67 seconds (about one minute, generally determined by the temperature difference of the vertical plate and nano-suspensions). For slow aggregation (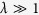), set *t*_*p*_ = 10^3^ seconds (16.67 min) and gives *λ* = 15.00. For fast aggregation (*λ* < 1), set *t*_*p*_ = 27.00 seconds (0.45 min), yield *λ* = 0.40.

**Figure 3:**
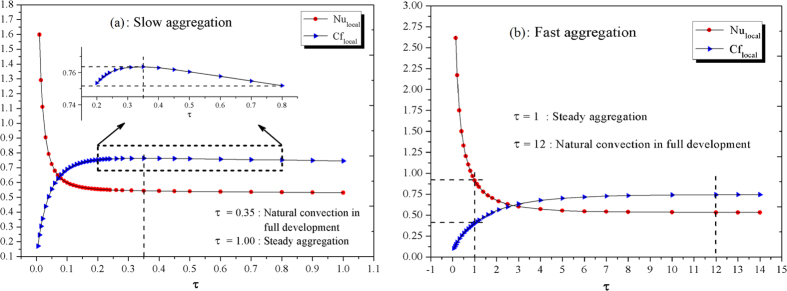
Different aggregation in convection dependence of time. (**a**) and (**b**) Both the local skin friction *Cf*_*local*_ and local Nusselt nnumber *Nu*_*local*_ versus demensionless time for slow and fast aggregation, respectively, with *ϕ*_*p*_ = 0.05 and *d*_*f*_ = 1.8 conditions.

We compute local skin friction *Cf*_*local*_ and the local Nusselt number *Nu*_*local*_ to confirm the time to reach full development of natural convection. Figure 3(a) shows the developing slow aggregation process over the time till *τ* = 1, and the local skin friction reaches a maximum (magnification figure inset Fig. 3(a)) at about *τ* = 0.35, i.e., *t* = 350 s (5.83 min), which manifests a full development of a convection flow. So the coupled development of both convection and aggregation together occurred in 0 ≤ *τ* ≤ 0.35. Subsequently, the dynamic aggregation plays its role continuously in duration 0.35 ≤ *τ* ≤ 1 to result in the decrease of local skin friction.

Figure 3(b) shows that the fast aggregation processes are in confinement 0 ≤ *τ* ≤ 1 with the initial development of natural convection. Subsequently, the convection, with an equilibrium aggregation (thermophysical properties will not alter any more), is ongoing to reach a steady state at around *τ* = 12, i.e., *t* = 324 s (5.40 min) and *Cf*_*local*_ and *Nu*_*local*_ are kept invariant. These results indicate that the unsteady thermal convection of a dynamic NS is apparently dependent on aggregation formation duration. SA makes the convection strong first (large *Cf*_*local*_) and then weaken, but convection is always growing to a steady state without weakening in FA.

**Figure 4:**
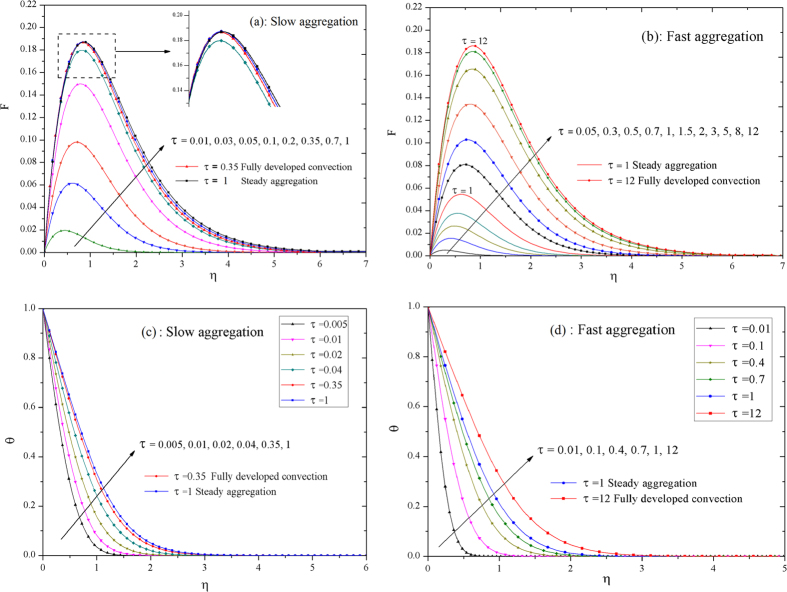
Velocity and temperature profiles by different aggregations for time. The convection velocity and heat transfer are coupled strongly in NCBL^29^,^30^ that NS undergoes the distinct development of thermal convection due to slow and fast aggregations, respectively. The dimensionless velocity field over time for slow aggregation case in (**a**) and for fast aggregation case in (**b**). The dimensionless temperature field for slow aggregation case in (**c**) and for fast aggregation case in (**d**), both of them with *ϕ*_*p*_ = 0.05 & *d*_*f*_ = 1.8 conditions.

The velocity distributions over time for the slow and the fast aggregation process with respect to convection are displayed in Fig. 4(a) and (b), respectively. As time goes on, the convection flow strengthens rapidly due to the thermal buoyancy force. For slow aggregation with fraction dimension *d*_*f*_ = 1.8 in Fig. 4(a), there are apparent intersection points (magnification figure inset Fig. 4(a)) between velocity profiles after the convection flow fully develops but before equilibrium aggregation (0.35 ≤ *τ* ≤ 1), i.e., the maximum of velocity do not change but its boundary layer thickens. Nevertheless, the convection velocity increase rapidly until the full development without any intersection points for fast aggregation in Fig. 4(b). The coupling development of velocity and temperature fields as an inherent feature in thermal convection system signifies the susceptible temperature field by different aggregation cases. Figure 4(c) and (d) illustrate the temperature distributions for slow and fast aggregation cases, respectively. The temperature increases rapidly over time, and the thickened temperature boundary layer shows that the heat transport from the vertical plate to the inner flow field is enhanced over the lifetime of the running system via incorporated impacts of heat conduction and convection simultaneously.

### The resultant of *ϕ*_*p*_ and *d*_*f*_

**Figure 5:**
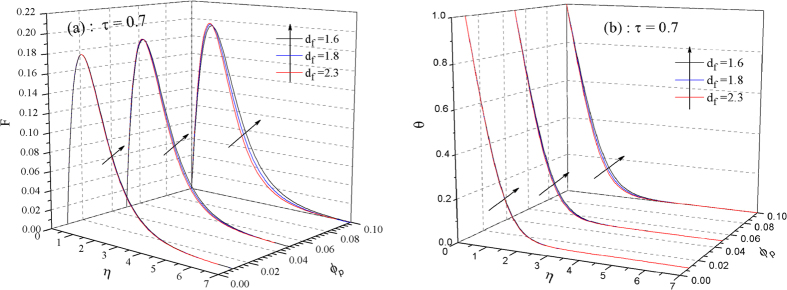
Volume fraction and fractal dimensions dependence of thermal convection. Synergistic effects of primary volume fraction of nanoparticles *ϕ*_*p*_ and fractal dimensions *d*_*f*_ on dimesionless velocity in (**a**) and temperature distributions in (**b**), respectively. The visible effects of *d*_*f*_ appear for concentrated nanofluid 0.05 ≤ *ϕ*_*p*_ ≤ 0.1, but negligible effects for dilute nanofluid 0.01 ≤ *ϕ*_*p*_ < 0.05.

Here, we focus on the discussions of slow aggregation case (a desired process in engineering applications). The time for the full development of convection is about *τ* = 0.35 for diffusion-limited aggregation *d*_*f*_ = 1.8, hence there is quite a long time period (0.35 ≤ *τ* ≤ 1) for non-equilibrium aggregation to affect the whole system. Here, we choose the time *τ* = 0.7, Figure 5(a) and (b) demonstrate the dependency of dimensionless velocity *F* and temperature *θ* distributions on fractal dimension *d*_*f*_ and primary volume fraction of nanoparticles *ϕ*_*p*_ together, respectively. The increment of *ϕ*_*p*_ over the range 0.01 ≤ *ϕ*_*p*_ ≤ 0.1 will give rise to the enhancement heat transfer (the increased temperature) as shown in Fig. 5(b), as well as a strengthen convection flow as shown in Fig. 5(a). In addition, results show that significant influences on both of *F* and *θ* with variation of *d*_*f*_ appear at high concentration 0.05 ≤ *ϕ*_*p*_ ≤ 0.1; in contrary, the effects in small magnitude are for dilute nano-suspensions *ϕ*_*p*_ = 0.01. As is well known, the larger fractal dimension the more random the aggregates is^34^, that is to say, for aggregates, the increased *d*_*f*_ manifests the more complicated aggregates in morphology. For concentrated NS, the increased *d*_*f*_, i.e., high complexity aggregation, not only can improve convection flow, but enhance heat transfer capability due to the thinner thermal boundary layer thickness shown in Fig. 5(b). Practically, one should adopt some measures to control aggregation in concentrated NS in order to maintain a system optimum working state. In general, some physical and chemical approaches, such as surfactant, ultrasonic vibration, temperature & pH control and particle surface charge modification, etc., can weaken aggregate processes^3^,^4^, namely, *d*_*f*_ is changeable for a certain concentration *ϕ*_*p*_ of NS. Obviously, it requires manpower and financial resources. Furthermore, if we define the energy efficiency (heat transfer efficiency) as the heat current absorbed by NS per unit area on the plate *q*_*w*_, then the increasing percentage of energy efficiency in micro devices using NS and base fluid is presented as 
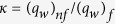
. For slow aggregation case, the enhanced energy efficiency *κ* using Al_2_O_3_/water nano-suspensions relative to the base fluid water for different primary volume fraction is shown in Table 2. The energy efficiency enhancement is prominent with the increase of *ϕ*_*p*_, particularly after the fully developed convection. However, the increased *d*_*f*_ can reduce *κ*, which we realize is important for the control of the aggregation processes. Although, the high volume fraction of NS can enhance energy efficiency, such NF can often cause the abrasion and blockage in micro devices due to the presence of numerous nanoparticles and clusters. On the other hand, a very dilute NS also can’t meet the requirements of energy efficiency enhancement. Consequently, the optimal options we suggest are at *ϕ*_*p*_ ~ 0.05 and we suggest to hold the diffusion-limited aggregation at *d*_*f*_ ~ 1.8.

### The variable momentum and enthalpy boundary layer thickness

Based on the theory of natural convection boundary layer (NCBL), momentum layer thickness (MLT) and enthalpy layer thickness (ELT) are primary characteristic values, whose expressions are given as^28^


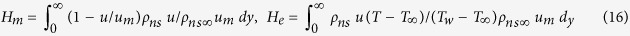


We substitute the similarity variables aforementioned into Eq. (16) with Boussinesq approximation and conventions 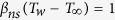 and 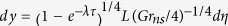 for simplicity^35^, yielding dimensionless momentum layer thickness *δ*_*m*_ and enthalpy layer thickness *δ*_*e*_, respectively:


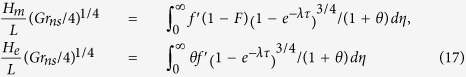


where 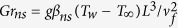 is Grashof number of nano-suspensions. In this approach, time-dependent fractal aggregation kinetics plays a critical role in unsteady NCBL. Therefore, *δ*_*m*_ and *δ*_*e*_ are evidently affected by time *τ*, volume fraction *ϕ*_*p*_ and fractal dimension *d*_*f*_ altogether.

**Figure 6:**
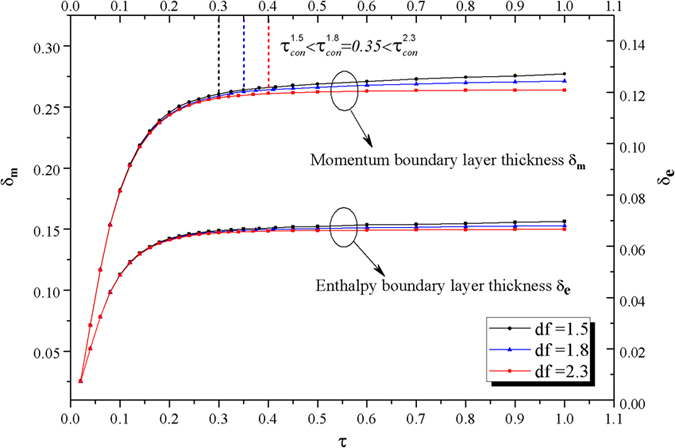
The growth of boundary layers thickness. Momentum and enthalpy boundary layers versus demensionless time for various fractal dimensions, and the numerical results demonstrate the time for fully developed convection with three different fractal dimensions that 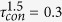, 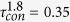 and 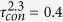.

In Fig. 6, one can readily observe the rapid growth of two layers in coupling development area, e.g., 0.01 ≤ *τ* ≤ 0.35 for *d*_*f*_ = 1.8. The increased *d*_*f*_ (in a proper range) makes both of the two layers thinner, but such effects are dominant after full development of convection, in particular, at a later stage of aggregation. Furthermore, the time *τ*_*con*_ for the full development of thermal convection can be extended with the increase of *d*_*f*_ in SA, i.e., 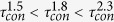 as shown in Fig. 6. All these results manifest the critical role of time-dependent fractal aggregation kinetics in NCBL.

**Figure 7:**
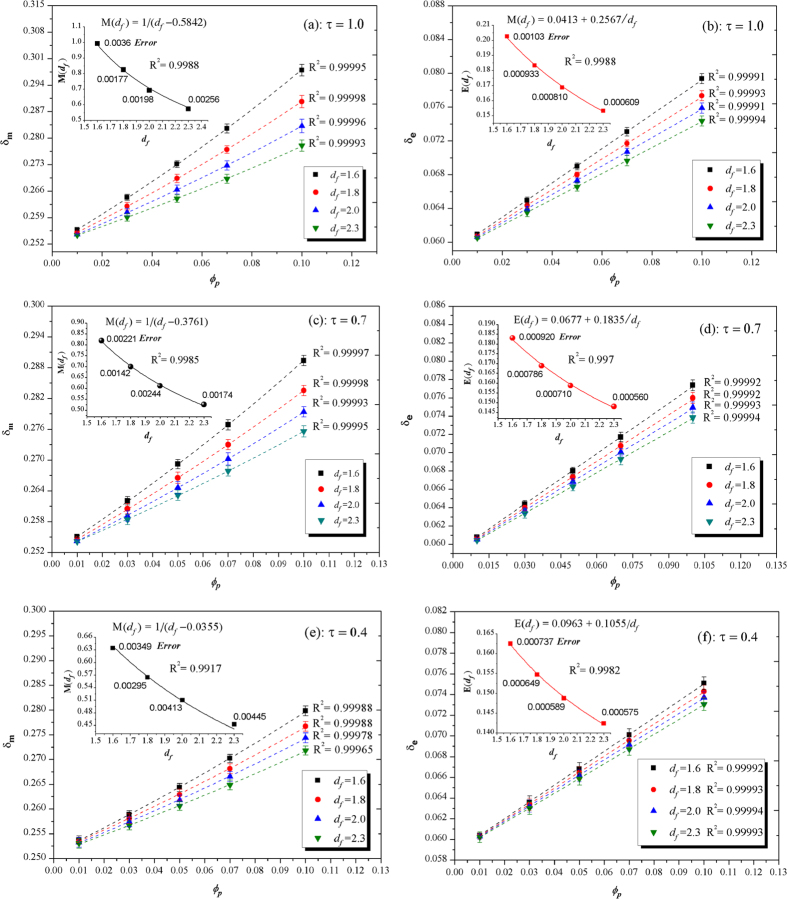
Boundary layer thickness fitting curves by fractal scaling relations. (**a**–**f**) The dimensionless momentum layer thickness *δ*_*m*_ and enthalpy layer thickness *δ*_*e*_ versus the primary volume fraction of nanoparticles *ϕ*_*p*_ for various time *τ*. The dashed lines are fitting results for *δ*_*m*_ and *δ*_*e*_ data with high degree R^2^ > 0.999. The solid lines in inside figures are also fitting results for *M*(*τ, d*_*f*_) and *E*(*τ, d*_*f*_) data, and all errors are the relative errors.

In addition to time *τ, δ*_*m*_ and *δ*_*e*_ are also evidently affected by *ϕ*_*p*_ and *d*_*f*_, which are displayed in Fig. 7(a–f). The dimensionless momentum layer thickness *δ*_*m*_ and enthalpy layer thickness *δ*_*e*_ increase largely with the increase of *ϕ*_*p*_, but the decrease of *d*_*f*_. Theoretically, the thinner boundary layer, the larger local Nusselt number *Nu*_*local*_, which signifies the enhanced convective heat transfer at the liquid-solid surface. For the fixed concentration *ϕ*_*p*_, the magnitude of the effects of *d*_*f*_ is gradually increased over time from *τ* = 0.2 to *τ* = 1 in the NCBL as shown in Fig. 7(a–f).

More importantly, we find a nonlinear growth of *δ*_*m*_, but a linear growth of *δ*_*e*_, with increased *ϕ*_*p*_ in the whole development process. The momentum boundary layer generated by the viscosity of fluids strongly depends on the enhanced effective viscosity of nano-suspensions in low-shear flow, e.g., natural thermal convection^29^,^30^. Numerous direct measurements of viscosity enhancement of hard sphere NS as the function of primary particles volume fraction exhibit a jamming transition near a random close packing (RCP) *ϕ*_*rcp*_ of hard sphere in low-shear flow, as a result, we logically infer an divergence packing friction in which the asymptotical infinite of momentum layer thickness is expected. The pioneering models for viscosity enhancement of NS suggested the divergence *ϕ*_*rcp*_ ≈ 0.64 with the optimal fitted values for data^36^,^37^,^38^ (also some model^39^ presented a value *ϕ*_*rcp*_ = 0.637 ± 0.0015). Here, we also take the RCP *ϕ*_*rcp*_ = 0.64 as a most probably divergence of *δ*_*m*_, while we know the possible maximum packing fraction is 0.7405 for general cases^40^.

**Figure 8:**
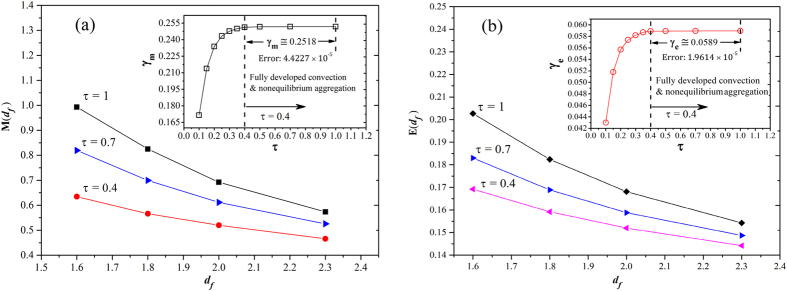
The dependence of correlation parameters on time. (**a**) The nonlinear sacling law of *δ*_*m*_, dependency of scaling exponent function *M*(*τ, d*_*f*_) on *d*_*f*_ for various time; Inset figure, scaling coefficient *γ*_*m*_ versus time, 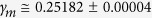 at fully developed convection but the non-equilibrium aggregation stage. (**b**) the linear sacling law of *δ*_*e*_, dependency of scaling exponent function *E*(*τ, d*_*f*_) on *d*_*f*_ for various time; Inset figure, linear scaling intercept *γ*_*e*_ versus time, 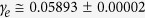 at fully developed convection but the non-equilibrium aggregation stage.

Consequently, we establish the nonlinear and linear fractal scaling relations for *δ*_*m*_ and *δ*_*e*_, respectively, as evidenced by the high-precision least square data fitting (R^2^ > 0.999) in Fig. 7(a–f):


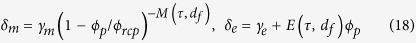


where, *γ*_*m*_ and *M*(*τ, d*_*f*_) are the scaling coefficient and the scaling exponent function for *δ*_*m*_, *γ*_*e*_ and *E*(*τ, d*_*f*_) are the scaling intercept and the scaling slope function for *δ*_*e*_. As an example for case *τ* = 0.7, we suggest *γ*_*m*_ = 0.25182 ± 0.000027 with *M*(0.7, *d*_*f*_) as a nonlinear function of *d*_*f*_ as shown in Fig. 7(c) which is the best fitted by simple form 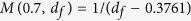 with error 0.0051, hence we conclude the scaling law as 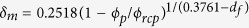. On the other hand, *γ*_*e*_ = 0.05893 ± 0.00002 is appropriate with *E*(0.7, *d*_*f*_) as a function of *d*_*f*_ as shown in Fig. 7(d), consequently, the scaling law is 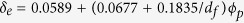. In fact, in Eq. 18, *γ*_*m*_ and *γ*_*e*_ are dependent on time, meanwhile 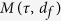 and 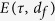 are also the functions of time and fractal dimension. A further probe on these considerations, Fig. 8(a) and (b) exhibits the dependency of scaling parameters on time and fractal dimension for *δ*_*m*_ (a) and *δ*_*e*_ (b), respectively. We find that 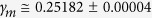 and 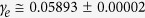 at fully developed convection regime (0.4 ≤ *τ* ≤ 1) for different fractal dimensions with almost no change over time, thus, these two scaling formulas can be reduced to simple expressions. Nevertheless, both *M*(*τ, d*_*f*_) and *E*(*τ, d*_*f*_) increase with time, in particular, *M*(*τ, d*_*f*_) is largely reduced in magnitude comparing to *E*(*τ, d*_*f*_) as *d*_*f*_ increases. It is a remarkable fact that the correlation parameters *M*(*τ, d*_*f*_) and *E*(*τ, d*_*f*_) as mentioned form earlier are failed to fit the data for the stage of unsteady convection (*τ* < 0.4, the primary stage of aggregation) by our repeated simulations. Therefore, an in-depth study is still essential in the future to derive the functions *M*(*τ, d*_*f*_) and *E*(*τ, d*_*f*_) to present the scaling laws more explicitly. At present, the fractal scaling relations Eq. (18) is more applicable for fully developed convection regime *τ* ≥ 0.4.

In conclusion, non-equilibrium aggregation as highlighted by its time-dependent and fractal behaviors play a critical role in the unsteady thermal convection boundary layer of the nano-suspensions. It can be categorized into two regimes, i.e., the slow and fast aggregation in terms of the thermal convection process using *λ* = *t*_*p*_/*t*_*m*_. Technically, we transform the original physical governing equations to the corresponding similarity equations by unique similarity variables which simplify solving complexity significantly and make the clear discussions consequently. For the slow aggregation, the aggregation (with certain fractal dimension value) reduces thermal convection due to the decrease of *Cf*_*local*_ and *Nu*_*local*_ after the flow fully develops. The increase of fractal dimension not only extends the time for fully developed convection, it but also reduces the thickness of the momentum and enthalpy boundary layers. In particular, the fractal scaling laws proposed by us on the basis of the data can well describe the dependence of *δ*_*m*_ and *δ*_*e*_ on both *ϕ*_*p*_ and *d*_*f*_ mathematically for the stage after a fully developed convection, in spite of the unsettled probe of fitting unsteady convection stage with primary stage of aggregation. Our results provide the theoretical perspective on the regimes how the dynamic NS induced by unsteady convection flow (shear-flow) exhibit the anomalous thermal conduction with effects of non-equilibrium aggregations, which is the convincing evidences in terms of phenomenological relations. In addition, leave unsettled whether the enthalpy boundary layers, unlike the momentum layer corresponding to the critical packing fraction due to the viscosity, are also divergent near the critical packing fraction of hard sphere particles in NS, which is indeed further research.

## Additional Information

**How to cite this article**: Sui, J. *et al*. Fractal aggregation kinetics contributions to thermal conductivity of nano-suspensions in unsteady thermal convection. *Sci. Rep.* **6**, 39446; doi: 10.1038/srep39446 (2016).

**Publisher's note:** Springer Nature remains neutral with regard to jurisdictional claims in published maps and institutional affiliations.

## Figures and Tables

**Figure 1:**
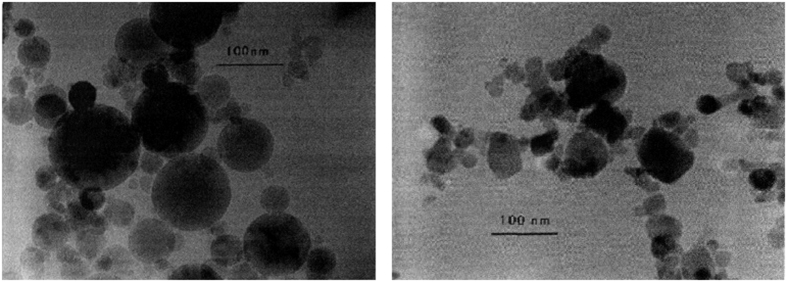
Aggregation and thermal conductivity predictions for stationary NS. Transmission electron micrographs (TEM) shows typical aggregated morphology in nanofluids: Al_2_O_3_/water (r = 19.2 nm, Left) and CuO/water (r = 11.8 nm, Right)^13^.

**Table 1:** The thermophysical properties of base fluid and nanoparticles^41^.

Physical properties	Water	Al_2_O_3_
*C*_*p*_ (**J**/**kg** **K**)	4179	765
*ρ* (**kg**/**m**^**3**^)	997.1	3970
*k* (**W**/**m** **K**)	0.613	40
*β* × 10^−5^ (**1**/**K**)	21	0.85

**Table 2:** Energy efficiency enhancement *κ* for Al_2_O_3_/water working nano-suspensions relative to the base fluid for slow aggregation in different time stages.

*Fractal dimensions*	*τ* = *0.2 Development of convection and aggregation processes*			*τ* = *0.4 Fully developed convection, but non*-*equilibrium aggregations*			*τ* = *0.7 Further evolution of aggregation processes*		
	*ϕ*_*p*_ = 0.01	*ϕ*_*p*_ = 0.05	*ϕ*_*p*_ = 0.1	*ϕ*_*p*_ = 0.01	*ϕ*_*p*_ = 0.05	*ϕ*_*p*_ = 0.1	*ϕ*_*p*_ = 0.01	*ϕ*_*p*_ = 0.05	*ϕ*_*p*_ = 0.1
*d*_*f*_ = *1.8*	102.11%	110.54%	121.04%	102.31%	111.53%	123.06%	102.58%	112.92%	125.88%
*d*_*f*_ = *2.0*	102.07%	110.30%	120.56%	102.22%	111.06%	122.08%	102.41%	112.03%	124.08%
*d*_*f*_ = *2.3*	102.01%	110.03%	120.00%	102.15%	110.52%	121.00%	102.22%	111.08%	122.13%
